# 

**Published:** 1964-04

**Authors:** 


### Contents

| | | |
|---|---|---|
| EDITORIAL . . . . . . . . . . . | | 25 |
| THE RISE OF OTORHINOLARYNGOLOGY. | *J. Angell James* | 26 |
| CONSCIOUS IMMOBILITY . . . . | *G. de M. Rudolf* | 35 |
| SOME NOTES ON ACUTE INTUSSUSCEPTION IN CHILDHOOD . . . . | *R. l'E. Orme* | 37 |
| A THERAPEUTIC TRIAL OF THREE PENICILLINS CONDUCTED IN A CASUALTY DEPARTMENT. . . . . . | *J. Woodyard et al.* | 50 |
| INTESTINAL RETICULOSARCOMA IN IDIOPATHIC STEATORRHOEA . | *Clinico-Pathological Conference* | 61 |
| CONFERENCE REPORT. . . . . . | *T. S. Wilson* | 68 |
| NOTES AND NEWS . . . . . . . . . | | 70 |
| OBITUARY . . . . . . . . . . . | | 70 |
| EXAMINATION RESULTS . . . . . . . . | | 70 |
| APPOINTMENTS . . . . . . . . . . | | 70 |

